# Non-indigenous species refined national baseline inventories: A synthesis in the context of the European Union's Marine Strategy Framework Directive

**DOI:** 10.1016/j.marpolbul.2019.06.012

**Published:** 2019-08

**Authors:** Konstantinos Tsiamis, Andreas Palialexis, Kremena Stefanova, Živana Ničević Gladan, Sanda Skejić, Marija Despalatović, Ivan Cvitković, Branko Dragičević, Jakov Dulčić, Olja Vidjak, Natalia Bojanić, Ante Žuljević, Marilena Aplikioti, Marina Argyrou, Marios Josephides, Nikolas Michailidis, Hans H. Jakobsen, Peter A. Staehr, Henn Ojaveer, Maiju Lehtiniemi, Cécile Massé, Argyro Zenetos, Luca Castriota, Silvia Livi, Cristina Mazziotti, Patrick J. Schembri, Julian Evans, Angela G. Bartolo, Saa Henry Kabuta, Sander Smolders, Edo Knegtering, Arjan Gittenberger, Piotr Gruszka, Wojciech Kraśniewski, Cátia Bartilotti, Miriam Tuaty-Guerra, João Canning-Clode, Ana C. Costa, Manuela I. Parente, Andrea Z. Botelho, Joana Micael, Joana V. Miodonski, Gilberto P. Carreira, Vera Lopes, Paula Chainho, Carmen Barberá, Rahmat Naddafi, Ann-Britt Florin, Peter Barry, Paul D. Stebbing, Ana Cristina Cardoso

**Affiliations:** a European Commission, Joint Research Centre (JRC), Ispra, Italy; b Institute of Oceanology “Fridtjov Nansen” – BAS, Varna, Bulgaria; c Institute of Oceanography and Fisheries, Šetalište I. Meštrovića 63, 21000 Split, Croatia; d Department of Fisheries & Marine Research (DFMR), Ministry of Agriculture, Rural Development and Environment, Cyprus; e Department of Bioscience, Aarhus University, Denmark; f Estonian Marine Institute, University of Tartu, Pärnu, Estonia; g Finnish Environment Institute, Marine Research Centre, Latokartanonkaari 11, 00790 Helsinki, Finland; h UMS Patrimoine Naturel (PATRINAT), AFB, MNHN, CNRS, CP41, 36 rue Geoffroy Saint-Hilaire, 75005 Paris, France; i Hellenic Centre for Marine Research, Institute of Marine Biological Resources and Inland Waters, GR-19013 Anavyssos, Greece; j Institute for Environmental Protection and Research (ISPRA), BIO-CIT, Lungomare Cristoforo Colombo n. 4521 (ex complesso Roosevelt), Località Addaura, 90149 Palermo, Italy; k Institute for Environmental Protection and Research (ISPRA), VAL-AMC, via Vitaliano Brancati 60, 00144 Rome, Italy; l ARPAE Emilia-Romagna SOD Daphne, Viale Vespucci 2, 47042 Cesenatico, FC, Italy; m Department of Biology, University of Malta, Msida, MSD2080, Malta; n Environment & Resources Authority, Malta; o Rijkswaterstaat, Water Transport and Environment, Ministry of Infrastructure and Water Management, Zuiderwagenplein 2, 8224, AD, Lelystad, the Netherlands; p Office for Risk Assessment and Research, Netherlands Food and Consumer Product Safety Authority, Ministry of Agriculture, Nature and Food Quality, Catharijnesingel 59 | 3511 GG | Utrecht, Postbus 43006, 3540, AA| Utrecht, the Netherlands; q Ministerie van Landbouw, Natuur en Voedselkwaliteit, Directie Natuur & Biodiversiteit, Cluster Marien, Postbus 20401, 2500 Ek Den Haag, the Netherlands; r GiMaRIS, Marine Research Inventory & Strategy Solutions, Leiden, the Netherlands; s Maritime Institute in Gdańsk, Department of Aquatic Ecology, Gdańsk, Poland; t Institute of Meteorology and Water Management – National Research Institute, Department of Oceanography and Baltic Sea Monitoring, Poland; u Portuguese Institute for Sea and Atmosphere, IPMA, I.P, Lisboa, Portugal; v MARE - Marine and Environmental Sciences Centre, Quinta do Lorde Marina, Sítio da Piedade, 9200-044, Caniçal, Madeira, Portugal; w Centre of IMAR of the University of the Azores, Department of Oceanography and Fisheries, Rua Prof. Dr. Frederico Machado, 4s, PT-9901-862, Horta, Azores, Portugal; x Smithsonian Environmental Research Center, 647 Contees Wharf Road, Edgewater, MD 21037, USA; y Centro de Investigação em Biodiversidade e Recursos Genéticos, InBIO Laboratório Associado, Pólo dos Açores, Universidade dos Açores, 9501-801 Ponta Delgada, Portugal; z Southwest Iceland Nature Research Centre (SINRC), Sandgerði, Iceland; aa Direção de Serviços de Biodiversidade e Política do Mar, Direção Regional dos Assuntos do Mar (SRMCT), Rua D. Pedro IV, 29, 9900-111 Horta, Açores –, Portugal; ab Directorate General for Natural Resources, Safety and Maritime Services, Avª Brasília, 1449-030, Lisboa, Portugal; ac MARE - Marine and Environmental Sciences Centre, Faculdade de Ciências, Universidade de Lisboa, Campo Grande, 1749-016, Lisboa, Portugal; ad Departamento de Biologia Animal, Faculdade Ciências da Universidade de Lisboa, 1749-016 Lisboa, Portugal; ae Center of Marine Research (Centro de Investigación Marina, CIMAR), University of Alicante, Carretera del Cabo de Santa Pola, 34, 03130 Alicante, Spain; af Swedish University of Agricultural Sciences, Department of Aquatic Resources, Division of Coastal Research, 74242 Öregrund; ag Centre for Environment, Fisheries and Aquaculture Science, Lowestoft, Suffolk, UK; ah Centre for Environment, Fisheries and Aquaculture Science, Weymouth, Dorset, UK; ai Institute of Biology Leiden (IBL), Leiden University, Leiden, the Netherlands; aj Department of Marine Zoology, Naturalis Biodiversity Center, Leiden, the Netherlands

**Keywords:** Alien species, Europe, Member State, Marine, Oligohaline

## Abstract

Refined baseline inventories of non-indigenous species (NIS) are set per European Union Member State (MS), in the context of the Marine Strategy Framework Directive (MSFD). The inventories are based on the initial assessment of the MSFD (2012) and the updated data of the European Alien Species Information Network, in collaboration with NIS experts appointed by the MSs. The analysis revealed that a large number of NIS was not reported from the initial assessments. Moreover, several NIS initially listed are currently considered as native in Europe or were proven to be historical misreportings. The refined baseline inventories constitute a milestone for the MSFD Descriptor 2 implementation, providing an improved basis for reporting new NIS introductions, facilitating the MSFD D2 assessment. In addition, the inventories can help MSs in the establishment of monitoring systems of targeted NIS, and foster cooperation on monitoring of NIS across or within shared marine subregions.

## Highlights

• Refined MSFD baseline inventories of non-indigenous species (NIS) are set in EU.
• The inventories are given per EU Member State (MS) and MSFD subregion up to 2012.
• The NIS lists provide a basis for reporting new NIS introductions in EU after 2012.
• Our work constitutes a milestone for the MSFD Descriptor 2 implementation.

## 1 Introduction

There are currently over 800 established non-indigenous species (NIS) in the European seas (Tsiamis et al., 2018), several of which exhibit invasive behavior and have a high impact on marine ecosystem services and biodiversity, causing adverse effects on environmental quality (Wallentinus and Nyberg, 2007; Katsanevakis et al., 2014; Ojaveer et al., 2015; Stæhr et al., 2016). Due to the threats they pose, there is an urgent need for appropriate management (Ojaveer et al., 2018). NIS occurring in the European seas are targeted in a series of legislative instruments, such as the European Union (EU) Biodiversity Strategy (EC, 2014) and the Marine Strategy Framework Directive (MSFD; EU, 2008, EU, 2010, EU, 2017). The MSFD requires EU Member States (MSs) to consider NIS when developing their marine management strategies, which aim to reach Good Environmental Status (GES) in European Seas.

NIS are treated as a distinct Descriptor (D2) of GES in the context of the MSFD (EU, 2017): “*Non-indigenous species introduced by human activities are at levels that do not adversely alter the ecosystem*”. The Descriptor D2 includes one primary criterion (D2C1), based on which “*The number of non-indigenous species which are newly introduced via human activity into the wild, per assessment period (6 years), measured from the reference year as reported for the initial asessment under Article 8(1) of Directive 2008/56/EC, is minimised and where possible reduced to zero. Member States shall establish the threshold value for the number of new introductions of non-indigenous species, through regional or subregional cooperation*”. There are also two secondary criteria of D2, dealing with the abundance and spatial distribution of NIS, particularly of the invasive ones, and their effects to indigenous species groups and broad habitat types (EU, 2017).

The environmental status of the European marine waters in the context of the MSFD was assessed by the MSs as part of the reporting obligations linked to the MSFD initial assessment, for most MSs in 2012 (hereafter referred to as the reference year). In that context, lists of NIS were reported in national level by each MS (hereafter referred to as initial reporting lists). These lists constitute the basis for the evaluation of the status of the pressure exerted by NIS in national marine waters and for reporting new NIS introductions.

Analysis of the initial reporting lists of NIS revealed important knowledge and data gaps, as well as vague definitions and significant differences on the level of detail and focus of the approach followed by each MS, pointing to the need for common standards (Palialexis et al., 2014). Moreover, these lists are currently considered outdated, as substantial changes in the status of several European marine NIS have recently occurred (Zenetos et al., 2017) as well as in-depth revision of the NIS introduction events data in some European regional seas (e.g. Galil et al., 2018; Ojaveer et al., 2017). For example, the non-indigenous status in Europe of many listed NIS has been challenged (e.g. *Hydroides dianthus*; Sun et al., 2017), while other species previously thought to be native are now considered as non-indigenous (e.g. *Cutleria multifida*; Kawai et al., 2016). In addition, recent studies, molecular findings in particular, have resulted in numerous changes in the taxonomic concept and nomenclature of many NIS, shedding light on their global native/non-indigenous biogeographic range (e.g. Bariche et al., 2015). As a result, several species were proven to be synonyms of other listed species, resulting in double inventory entries (e.g. *Anotrichium okamurae* is now considered to be a synonym of *A. furcellatum*; see Guiry and Guiry, 2018). Following these developments, it is necessary to revisit and revise the initial reporting lists of NIS of the MSs.

Consequently, in order to ensure the consistent and comparable implementation of the MSFD D2 it is expedient to acquire consolidated and refined baseline inventories of NIS per MS and marine MSFD subregion. The current paper aims towards the fulfillment of the above target and provides the starting point information, facilitating discussions at national, regional and inter-regional levels, which could help in developing recommendations and best practices for common agreed methodological standards and rules for setting reference values for the number of new NIS introductions in the context of the MSFD, and particularly of D2C1.

## 2 Materials and methods

The refined baseline inventories provided in this paper are based on the best available knowledge arising from an assessment of the initial reporting lists of NIS for the MSFD (mostly in 2012) and the updated data from the European Alien Species Information Network (EASIN, 2018), in collaboration with the national experts appointed by the MSs competent authorities being responsible for the MSFD D2 implementation.

### 2.1 Initial MSFD lists compared with EASIN

The initial reporting lists were compared and analyzed with the updated information found in EASIN, managed by the European Commission (Katsanevakis et al., 2012; Deriu et al., 2017). EASIN offers a dynamic inventory of NIS that is continuously updated, revised and validated through a process, which includes several steps aimed at pursuing high quality standards, with the engagement of external experts and the EASIN Editorial Board (Tsiamis et al., 2016). As a result, EASIN follows the latest scientific findings about NIS in Europe. The pan-European inventory of EASIN used for the current study was updated up to 2017 (EASIN Catalogue version 5.6). In order to ensure comparison during the same timeframe, i.e. species found in each MS by the reference year of MSFD (in particular up to 31.12.2011) EASIN data were filtered by date. In that way, NIS recorded in EASIN but found in 2012 or afterwards in each MS was not considered.

The aim of this comparison was to detect species incongruities. Mismatches could correspond to: a) NIS within the initial reporting lists that are nowadays considered as native in whole Europe; these species were proposed for exclusion; b) NIS within the initial reporting lists with outdated nomenclature or bearing typos; the most up to date nomenclature based on WoRMS (2018) and correct orthography were indicated; c) NIS which had been omitted from the initial reporting lists, based on EASIN spatial data up to 31.12.2011; d) NIS within the initial reporting lists that, after careful checking, were excluded due to erroneous historical information and/or insufficient evidence (e.g. Ojaveer et al., 2017).

EASIN distinguishes NIS (marked as “alien” in EASIN website) from cryptogenic, i.e. species with no definite evidence of their native or non-indigenous status (due to unknown origin or due to unclear mode of introduction from native range: natural spread vs human mediated), and from questionable species, i.e. NIS with insufficient information or new entries not verified by experts or NIS with unresolved taxonomic status. On the other hand, the vast majority of MSs did not make a related distinction in the initial reporting lists. Several species listed in the initial reporting lists were matched with EASIN, but they were tagged in the latter either as cryptogenic or questionable species. These species were highlighted for the attention of MSs appointed experts. In addition, following the recent terminology proposed by Essl et al. (2018), we replaced the term “questionable” by the term “data-deficient”.

For our analysis we generally did not take into consideration oligohaline (estuarine) and freshwater NIS. However, the above NIS were included from several MSs when these species have been also found (even occasionally) in their marine waters. These entail mostly the oligohaline and freshwater species found also in the low saline Baltic Sea. When it comes to parasitic NIS, these were omitted since from a legislative perspective they are managed under the Aquatic Animal Health Directive (2006/88/EC;EU, 2006) rather than the MSFD. All results were compiled in an excel file, which was provided to the experts for checking and validation.

### 2.2 Member States appointed experts checking

Appointed experts from the EU MSs were invited to check and validate the comparison assessment between the initial reporting lists of NIS and the EASIN data, and to supplement it with national data. In this way, any error and omission could be addressed for each country. Several scientific information sources were used by the MSs. AquaNIS (2018), which is routinely updated by the ICES Working Group of Introduction and Transfers of Marine Organisms (WGITMO), substantially contributed to this phase when it comes to the Baltic Sea and several NE Atlantic countries. In more detail, the MSs experts were invited to:

a) Assess the validity of the proposed exclusions for NIS that are considered nowadays as native in Europe or were proven to be historical misreportings. In addition, experts commented on cryptogenic and data-deficient species. The occurrence of each species per national MSFD subregion was also indicated.
b) Assess the validity of including previously omitted NIS into the refined MSFD baseline inventories up to the reference year. Experts included also omitted cryptogenic and data-deficient species. The presence of each NIS per national MSFD subregion was again indicated.

**Fig. 1:**
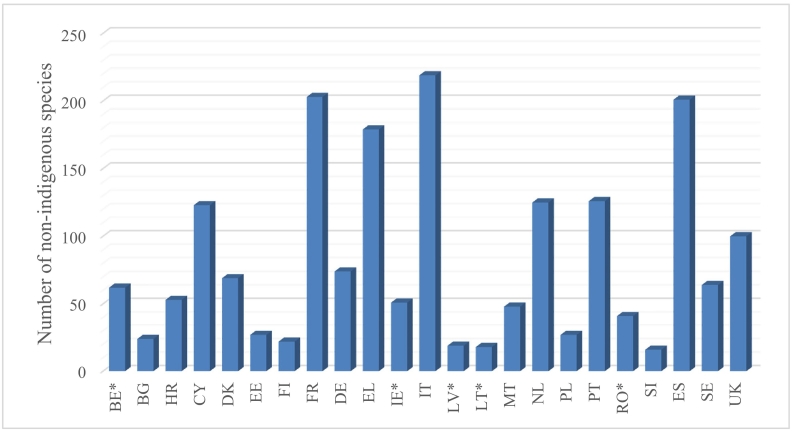
Refined total number of non-indigenous species up to 31.12.2011 per EU Member State. For Member States with an * data are exclusively based on the comparison assessment between the initial reporting lists of NIS and EASIN.

National experts from 16 of the 23 EU MSs with marine waters provided feedback and additional data, and have endorsed the final output (Fig. 1). Moreover, Spain partially contributed and was able to provide final endorsement for species found in the Spanish Western Mediterranean Sea. Germany also partially contributed but was unable to conclude in a final endorsed list of NIS. The remaining 5 MSs did not provide feedback, highlighting problems such as time limitations, or lack of monitoring and updated data since the reference year of the MSFD. For these countries, the refined baseline inventories provided in the current paper are based exclusively on the comparison assessment between the initial reporting lists and the updated EASIN data. For these countries, the baseline inventories should be considered as the best available knowledge, in the absence of related information checked and validated by national experts.

The checking phase promoted collaboration and coordination with MSs experts and ensured data sharing and exchange, leading to consolidated and refined MSFD baseline inventories of NIS by the reference year of MSFD per country. In these lists cryptogenic and data-deficient species were also included. The refined baseline inventories of NIS at MSFD subregion level were built based on the merging of the national inventories, according to the related subregion. In that phase only NIS were considered, while cryptogenic and data-deficient species were skipped due to their high uncertainty.

## 3 Results

In total, 787 non-indigenous taxa have been found in EU marine (Macaronesia included) and, in some cases, also in transitional waters up to 31.12.2011. These include 139 Macrophytes, 125 Mollusca, 125 Arthropoda, 120 Chordata, 98 Annelida, 39 Bryozoa, 35 Cnidaria, 15 Porifera, 14 Foraminifera and 77 taxa belonging to other taxonomic groups (Appendix 1). *Magallana gigas*, *Ficopomatus enigmaticus* and *Codium fragile* are the most widespread NIS across EU marine waters, found in 9 MSFD subregions, followed by the species *Acartia* (*Acanthacartia*) *tonsa*, *Callinectes sapidus*, *Hydroides elegans*, *Melanothamnus harveyi* and *Rapana venosa*, which have been found in 8 subregions. On the other hand, more than half NIS (480 taxa) have been reported by only one marine MSFD subregion.

The refined baseline inventories of NIS, cryptogenic and data-deficient species per MS are given in detail in Appendix 2. The total number of NIS (excluding cryptogenic and data-deficient species) per MS is displayed in Fig. 1, showing the highest values in Italy, France, Spain and Greece, while the lowest in Slovenia, Lithuania, Latvia and Finland.

**Fig. 2:**
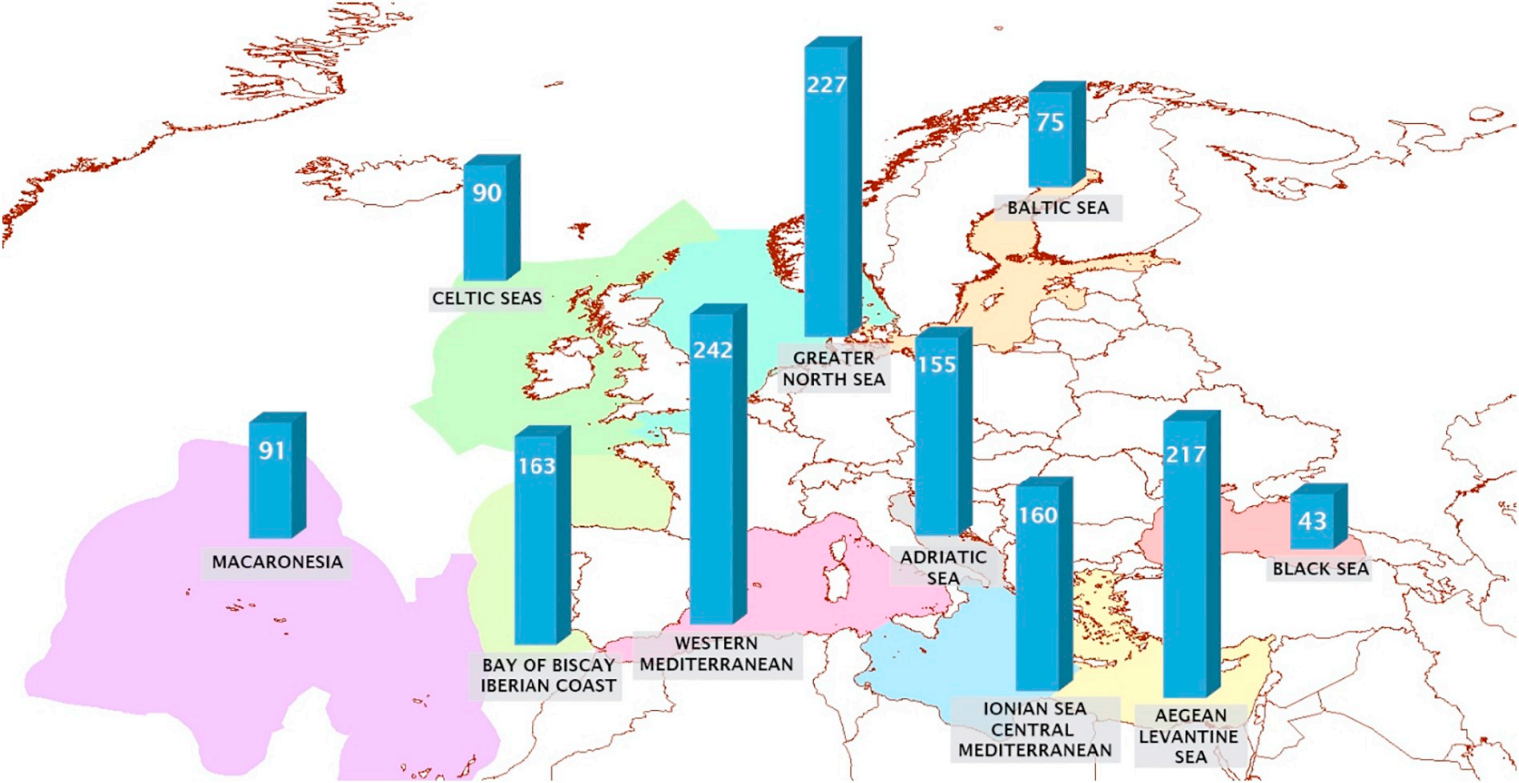
Refined total number of non-indigenous species up to 31.12.2011 per EU MSFD marine subregion. The graphs represent species reported only from EU countries (not including data from non-EU countries).

The total number of NIS per MSFD marine subregion is given in Appendix 1 and displayed in Fig. 2. The highest numbers of NIS have been reported from the Western Mediterranean Sea, the Greater North Sea and the Aegean-Levantine Sea, with the lowest numbers from the Black and the Baltic Seas.

**Fig. 3:**
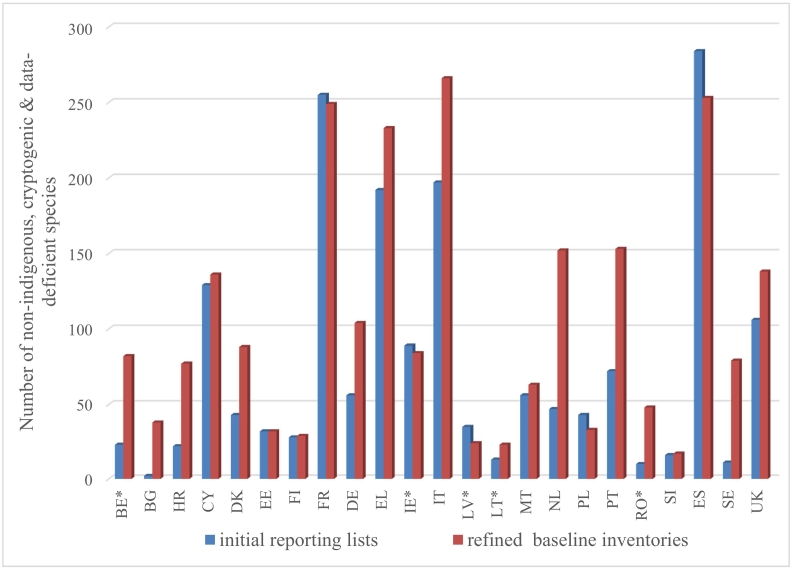
Total number of non-indigenous, cryptogenic and data-deficient species by the reference year of MSFD per EU Member State, based on both initial reporting lists (blue) and refined baseline inventories (red). For Member States with an * the refined baseline inventories data are exclusively based on the comparison assessment between the initial reporting lists and EASIN. (For interpretation of the references to colour in this figure legend, the reader is referred to the web version of this article.)

Finally, the comparison between the total number of NIS, cryptogenic and data-deficient species per MS in the initial reporting lists and their numbers in the refined baseline inventories revealed that the number of species had been strongly underestimated for most MSs in the initial reporting (Fig. 3). In overall, 40 species were missing on average per MS from the initial reporting lists, representing about 38% of the total number of species within the refined baseline inventories. On the other hand, 12 species per MS were excluded on average from the initial reporting lists (16% of the total number of the initially reported species).

## 4 Discussion

It is generally agreed that within the MSFD priority should be given to prevention of new NIS introductions (EU, 2010, 2017). Therefore, the evaluation of trends in new NIS introductions can reveal valuable information to support NIS management, in particular to reduce the risk of new introductions through the management of their pathways. The refined baseline information produced in the current paper is essential for the detection of new introductions in the context of the MSFD criterion D2C1 (EU, 2017), and its coherent assessment within and across the marine MSFD regions and subregions. Such refined baselines are not only important regionally (i.e. for the EU and Europe), but assist to locate EU into a much broader geographic scale, essentially under the current global trend of increasing human impact on marine ecosystems and opening totally new introductions gateways (e.g. shipping activities due to climate change in the Arctic; see Chan et al., 2018). While MSs have developed national monitoring programmes under the MSFD, there would be clear benefits from effective and harmonized NIS monitoring methodologies (Zenetos et al., 2009; Lehtiniemi et al., 2015).

Nominated experts of the MSs have played an active role in refining the baseline inventories by substantially contributing with national data, validating and endorsing the updated information based on EASIN, and thereby assisting in reaching to harmonized assessments in terms of some critical information (e.g. species status). The outcome revealed that the number of NIS had been strongly underestimated for most MSs in the initial reporting. Although about 16% of the species initially listed were excluded from the refined baseline inventories, a significant portion of species (about 38%) was missing from the original reporting lists delivered in 2012. This may be the result of improved reporting processes being established within MSs subsequent to the implementation of the MSFD, that has resulted in the reporting of species detected prior to 2012. However, it is likely that as monitoring and sampling efforts increase in MSs, species introduced (but not detected) prior to 2012 will be detected and potentially reported as new introductions.

Most NIS have been recorded in Italy, France, Spain and Greece, while the fewest have been found in Slovenia, Lithuania, Latvia and Finland. These differences likely reflect factors such as the coastline length of each country, monitoring efforts, and the density of gateways and pathways in certain marine subregions (see also Nunes et al., 2014; Tsiamis et al., 2018).

Concerning the subregional scale, most NIS have been reported from the Western Mediterranean Sea, due to the involvement of three countries (parts of France, Spain, Italy) with long coastlines, intense sampling effort and high intensity of certain pathways (shipping, aquaculture; Katsanevakis et al., 2013, Katsanevakis et al., 2014). In the Aegean-Levantine Sea there are fewer NIS than the Western Mediterranean Sea, despite the high numbers of Lessepsian NIS occurring in the former (Zenetos et al., 2012; Galil et al., 2017). This is because only two countries were taken into account (Greece, Cyprus), while all other Levantine countries were not considered since they fall outside the EU. The same reason can justify the very low number of species in the Black Sea, since only two Black Sea countries were taken into account (Romania and Bulgaria). In addition, the relatively low number of NIS reported from Macaronesia is most likely an underestimation, attributed to monitoring gaps.

During the compilation of the refined baseline inventories, a large number of cryptogenic species was observed. This was due to the high uncertainty regarding the origin, biogeography, pathway and consequently the non-indigenous status in Europe of several marine species, such as *Palaemon elegans* in the Baltic and *Antithamnionella spirographidis* in the Mediterranean Sea (Verlaque et al., 2015; Cross et al., 2016; Lasota et al., 2016). The uncertainty regarding the native vs non-indigenous status is even higher when it comes to the unicellular planktonic species (see also Gómez, 2008). As a result, there was high variance of the number of unicellular planktonic species included in the inventories among the MSs, even between neighboring countries, reporting either long lists of them or just a few. We would suggest that unicellular plankton NIS should be treated with caution (e.g. flagged with high uncertainty) until further research clarifies their enigmatic status.

Another issue was the oligohaline species. In our analysis we have excluded oligohaline NIS, unless these have been also found (even occasionally) in marine waters. Indeed, Baltic Sea countries have included several of them since these species can be also found in the low salinity of the Baltic Sea. Nevertheless, these species might be present in other MSs as well, but they were not listed in their inventories since they occur exclusively in their inland systems (e.g. *Corbicula fluminea*, *Cordylophora caspia*, *Elodea canadensis*, *Elodea nuttallii*). In overall, more work on oligohaline NIS is required in order to ensure that these species can be addressed in a fully consistent way at EU level.

Finally, there is a need of coherence among MSs regarding the monitoring effort on specific taxonomic groups, e.g. data gaps in jellyfish monitoring in Denmark. Consequently, the endorsement of similar monitoring schemes and guidelines on how to determine the non-indigenous status of specific species groups is essential. The network of MSs experts has the capacity to work on these issues and lead the way forward to a more consistent methodology regarding the NIS considered in terms of the MSFD.

## 5 Conclusion

Our paper constitutes a milestone for the overall MSFD D2 implementation, derived from a joined work of the appointed D2 experts of the majority of the MSs. It contributes to establishing refined baseline inventories of NIS up to the year of the initial assessment of the MSFD, which is the basis for the assessment of the primary criterion D2C1. It is a prerequisite for setting a baseline for this criterion, allowing for the determination of the number of new introductions subsequent to 2012 per MS. In addition, the refined inventories of NIS could support the process towards the establishment of the threshold values for D2C1 (i.e. the number of new introductions of NIS which reveals GES at regional or subregional level), through the information related with the time trends of the listed NIS introductions and their associated pathways. In general, the refined baseline inventories can help MSs in the establishment of surveillance systems of targeted NIS, such as those with invasive behavior addressed in the secondary criteria of D2, and could foster MSs cooperation and coordination across or within shared marine subregions.

However, our work also highlights the uncertainty on several species across EU marine waters, especially when it comes to the non-indigenous status of several species. Moreover, without a standardized monitoring framework, addressing D2C1 is even more challenging. The implementation of MSFD D2 requires further work and needs further support. The on-going work in the MSs, Regional Sea Conventions and projects will provide experience and knowledge, which should feed in the implementation process. There is a need for harmonization and coherent implementation of MSFD D2, mostly in relation to NIS reference points, monitoring, and thresholds. The current work is undoubtedly setting an important and scientifically validated cornerstone towards achieving these high-level objectives.

The following are the supplementary data related to this article.

Appendix 1 Non-indigenous species found in EU marine waters up to 31.12.2011. The presence at the subregional level of the Marine Strategy Framework Directive as well as the taxonomic information is provided for each taxon. Appendix 1

Appendix 2 Refined baseline inventories of marine non-indigenous, cryptogenic and data-deficient species per EU Member State in the context of the Marine Strategy Framework Directive (MSFD), found in each country up to 31.12.2011. The presence of each taxon in the subregional level of the MSFD is also marked per Member State. Appendix 2
